# Some Scientific Conclusions Concerning the Vice Problem*Read before the Seventh International Purity Congress, at Minneapolis, Minn., Nov. 7-8, 1913.

**Published:** 1914-03

**Authors:** T. D. Crothers

**Affiliations:** Superintendent Walnut Lodge Hospital, Hartford, Conn.; President New York Medicolegal Society, Etc.


### TEXAS MEDICAL JOURNAL

Established July, 1885

F. E. DANIEL, M. D., - - - - - Editor, Publisher and Proprietor

Published Monthly.—Subscription, $1.00 a Year.

VOL. XXIX. AUSTIN, MARCH, 1914. No. 9

The publisher is not responsible for the views of the contributors.

### Original Articles.

For Texas Medical Journal.

### Some Scientific Conclusions Concerning the Vice Problem.*

BY T. D. CROTHERS, M. D., SUPERINTENDENT WALNUT LODGE HOSPITAL, HARTFORD, CONN.; PRESIDENT NEW YORK MEDICO-LEGAL SOCIETY, ETC.

This congress is a distinct recognition of the cosmic consciousness of the gravity of a condition that is perilous to the progress of the race. The social evil cannot be properly studied, or understood from a theoretic or moral point of view, because it is a physical and mental degeneration, a veritable insanity.

Every advance of science reveals more clearly the possibilities of checking, breaking down and stamping out these degenerative forces, the same as infectious diseases; also that it is a field of preventive medicine which, when recognized and occupied by scientific men, will revolutionize the present race march.

### WHAT IS THE SOCIAL EVIL?

The answer which science makes to this question is, that it is the direct result of a widespread delusion and obsession, an insane impulse for the unrestrained gratification of the sexual functions of the body; an obsession that dominates every consideration of duty and obligation and purpose in life, in which procreation is not considered.

This delusion has materialized into a business, based on the theory that the sexual impulse demands gratification, and up to a certain limit is not deleterious. An extensive literature has

*Read before the Seventh International Purity Congress, at Minneapolis, Minn., Nov. 7-8, 1913.

grown up about this delusion, invested with prestige, custom and theory, that have no support from modern, scientific studies, and are literally projections of savagery and barbaric conceptions of life, fostered and retained in the present.

It is literally the Bushman's instinct which seeks by force to gratify every impulse, regardless of conditions and at all hazards.

Scientific studies show clearly that women as a class are less inclined to lives of immorality than men, but as slaves they have been degraded through the ages, and much of their higher instinct has been suppressed, and they have been made victims and prey of others.

Science also indicates that the army of women prostitutes are of a higher type morally and mentally than the men who sustain and support them; also that men are the real prostitutes and degenerates, and women are the slaves and victims.

There is no function of the human body about which has gathered so many delusive theories, stupid misconceptions and actual insanity as the sex organs.

### MAGNITUDE OF THE SOCIAL EVIL.

The assertion is sustained by a great variety of statistical studies that the number of women who can be called prostitutes in this country exceed one million, and that the number of men who sustain and support them extends far into the millions. Whether they are increasing or diminishing is a startling question, which is answered both in the affirmative and negative by different authors, in various parts of the country.

The white slave traffic has received striking confirmation of its extent in the statement of Mr. I. C. Baker, Secretary of the Travelers' Aid Society of New York, that over 50,000 girls and women disappear annually in this country, and are so completely lost that no trace is found of them by their families or friends.

Seventeen hundred girls and women disappeared in 1910 while journeying between the cities of Chicago and New York. In 1912 six hundred dropped out of sight of this route, showing a change for the better through the efforts of the Travelers' Aid Society and others. During the last ten years this society reports the rescue and assistance of over seventy thousand girls. These figures are unmistakable indications of the extent of this evil in only one particular. Practical observation in every city and town of the country gives some idea of the magnitude and presence of this great problem.

### SOME OF THE PROMINENT CAUSES.

Science asks the question: Why should these armies of dissolute men and women exist? and answers in part, that they are the result of distinct growth, soils and surroundings, also that they are governed by positive laws of dissolution which move with absolute certainty. This can be verified by studies of heredity, alcoholism, ignorance, bad living, bad training, and bad surroundings.

In the world of science there are no chance happenings. Disseases and degenerations all have distinct causes and follow distinct lines. The vice evil is no exception. Prostitution in men and women, in a very large percentage of cases, is traceable to hereditary defects.

A study of the laws of growth show that human, animal and plant life follow each other in a continuous march, reproducing the exact conditions of the ancestors, with slight variations here and there. The boy or girl is to a large degree the exact reproduction of the parents, with additions and subtraction from the ancestral types. Parents who are defective, immoral, insane and inebriates, and paranoiacs of all grades, cannot have healthy, normal children. They will be defective, abnormal and degenerate in some way sooner or later.

Eugenic studies support this with an enormous mass of evidence, sustained by the experience of stock breeders, agriculturists and horticulturists. Studies of families extending over two or three generations are often startling in the confirmation of these facts.

Individual studies of prostitutes show that from 50 to 75 per cent are descendants of diseased and immoral parents. They are defectives, degenerates, incapable of living normal lives, hence become abnormal, with or without any special exciting causes. There is a very extensive literature confirming this, with illustrations in almost every community.

*Alcohol.*—Next to heredity, alcohol is a most prominent, exciting and predisposing cause of the social evil. Research shows that the particular parlyzing action of alcohol on the higher brain functions lowers the consciousness of right and wrong and obscures the reason, lessening the power of control, and making the victim susceptible to the very basest impulses of life. Dissolute men and women recognize this fact and use alcohol to weaken the caution and reason of the individuals they wish to influence. Thus alcohol becomes almost inseparably connected with prostitution, immorality, and conditions that lead up to it. In reality, alcohol produces a distinct form of moral paralysis. This palsy of the higher faculties, traceable to alcohol, extends to the next generation. Numerous illustrations and an increasing mass of evidence sustain this fact.

The following is an incident: In some studies of 200 families in Switzerland, it was found that over 80 per cent of the first-born children were inferior and showed unusual abnormalties of mind and body. Those born later were far superior, physically and mentally. The explanation was found in the fact that the parents at the wedding and for a week or two afterwards celebrated their nuptials by prolonged drinking of spirits and other excesses. The children born during this period were very largely defective and degenerates. Many of them were feeble-minded, prostitutes, criminals and mentally diseased. The children born at a later period, when the parents were abstainers, were of a much higher type, stronger and in every way superior. This study is confirmed by many examples. Thus parents who drink wines and beers at the table, and then later abstain, are astonished to find that the children born during the wine-drinking period show greater weakness of morals, mind and body and are degenerates in later life.

A common illustration is that of persons who become wealthy, and add to their menu as a luxury wines, etc. Later they realize the fact that children born during their prosperous period are weaker than those born before.

A recent study points out the fact that many degenerates, immoral men and women possessing wealth have been petted children and given wine and spirits in early life. Alcohol literally furnishes a favorable soil for the growth of sexual excesses and general degenerations.

Fast men and women who suddenly become so from surroundings are types and illustrations of alcoholic dementia and palsy, beginning at home and extending through college life.

The moderate drinkers, or the persons who use spirits and beer daily and are never seen intoxicated, furnish a large element for the promotion and practice of vice. Their consciousness of duty and responsibility is clouded and their brain activities are lowered and anesthetized; they are victims of every impulse of the body, hence furnish a large proportion of vice adherents.

The spasmodic drinker, who during his drink excess displays moral dementias and sexual deliriums, is anesthetized and the subject of morbid impulses which drive him to the lowest excesses. In reality, alcohol of all drugs, whose physiological action has been studied, has the most positive destructive power, breaking up conduct, character and conceptions of duty.

*Moral Insanity.*—The study of prostitution along exact lines, extending to the origin, growth and development, shows it is a moral insanity, distinct and positive as any other disease of the brain. The real central governing power of the nerve force and control is paralyzed. The personality or character of the person is the consciousness of his relations to life, duty and obligation to others and the object and purpose of existence. This is the highest and last formed function of the brain and nervous system, and is often the first to break down. Injury of this faculty is marked by morbid impulses, abnormal conduct, feebleness of control, and general degeneration.

The kleptomaniac is a moral paretic. He has lost respect for the rights of others to property; he has lost his sense of duty and obligation, and is dominated by selfish impulses to possess what is not his own. The dipsomanic is another, whose insane thirst for spirits destroys his consideration for himself or others. He has only one thought—that of securing the poison. Erotomania is another form of moral paresis which is more common among men than women. The defenders and promoters of fast life and prostitution are dominated by insane sexual impulses, the gratification of which is the center of all their thoughts and activities.

Immoral women recognize this and seek remuneration, control and dominance over such persons. There is perversion of reason, failure to recognize any other consideration and disposition to sacrifice everything in the present and future for the gratification of this one impulse. There is associated with this irresponsibility and reckless disregard of the energies of the body.

There are false reasonings, distinct hallucinations and destructive delusions, and, with all these, a fatalism that sinks the person to the level of the brute. Individual rights and property that are obstacles to this impulse are unrecognized.

Literally, the words insanity and moral paresis describe more distinctly than any other the exact mental condition of the person. In other respects there is judgment, reason and recognition of the surroundings. The mentality displayed is to explain, apologize and indicate that this conduct is normal, reasonable and right. It is a mental, moral and physical suicide in the broadest sense of these words.

*Ignorance and Environment.*—There is a very wide range of active and exciting causes of immorality due to ignorance and surroundings. In all probability, ignorance is the primary factor in all cases, from the most highly educated down to the lowest grade of intelligence. This want of knowledge relates to sex life, sex purposes, sex activities and sex control, and is hedged about with most dangerous theories, misconceptions and delusive reasonings concerning the individual, his life and position in the race march. The children of the slum districts are brought up to regard prostitution as a normal condition. Children of the wealthy and educated have much the same idea, with the addition that it brings a certain pleasure, and is a part of the luxury of an ideal life.

Thus education, culture and the absence of it contribute markedly to the promotion of immorality.

With this there is intense selfishness to procure gratification of the impulses of the body at all hazards. The strains and drains of life destroy the ethical notions of duty, and poverty, bad living, defective nutrition, delirium for wealth, position and luxury are all favorite soils for the growth of prostitution.

False theories, false ideals of life require only bad surroundings and conditions to materialize into bad lives, disease and early death. In all circles of life there are innumerable armies of men and women who are unable to adjust themselves to the present conditions and are unable to judge of the effects of certain causes, hence they are victims of morbid impulses of both body and mind, which drive them into tides of degeneration and dissolution.

There can be no doubt that poverty, excitement, temptations, bad surroundings, all contribute to what is called the white slave traffic, but the real causes are farther back. They are mental and physical defects inherited and acquired, want of culture, profound ignorance, ambition, or purpose, with no capacity to resist or understand the meaning of temptations and the possibilities of their own laten powers.

Thus an army of degenerates who are ignorant and have defective training, when placed in bad surroundings, both mental and physical, the results will be immorality in every form and degree. This is the direct law of cause and effect, to which there are no exceptions.

### NATURE'S METHODS OF ELIMINATION.

The fact is seldom recognized that nature is continuously making great efforts to check and destroy prostitution. Immorality is followed by disease, virulent, destructive, and transmissible. Sexual excesses are always followed by loss, failure, feebleness, and eliminative diseases, which destroy the victim. Prostitutes invite an early death, have diminished longevity, and are continuously struggling against the eternal law of elimination.

Venereal diseases are the direct penalty for violation of the sex laws. Disease processes are created which go on through the second and third generation in a continuous dissolution, unless checked by new energies and forces. Syphilis is one of these diseases. The individual and the race suffering from this affection is literally turned from the main line on to a side track that leads to early death. The central purpose of life, to transmit to the future the best vigor and force of the present, has been checked and diverted, and permanently altered. While the process of degeneration may be suspended and halted for a time, there is a fierce Nemesis that never fails to punish to the last degree the violation of these laws.

There are infectious inflammations common to sex excesses that are equally destructive in their action, and are transmitted with virulence and persistency uncommon to other diseases.

The almshouses, the hospitals, the insane asylums and other places are filled with victims of these diseases whose degenerations began by violations of the sex laws, and whose present condition is nature's punishment and nature's methods of elimination. Before nature's tribunal there are no compromises, no pardons, no suspended sentences, but absolute, positive punishment with destruction and mortality of the poor victim.

The medical profession spend much time and effort trying to discover agents that will lessen the severity of these diseases and restore the victim to health. They succeed in a small degree, but the degenerative processes go on, ever ready to break out in some unknown form or direction.

If persons were educated to know the great central object of life, the transmission of the present to the future and the physiological and psychological purposes of the sex organs, and the power of control, there would be no venereal diseases.

These maladies are a reflection on the intelligence of the victims, and shows their barbaric ignorance. All scientific studies show that these diseases are in the same family groups as contagions, plagues, scourges and other infectious diseases; that they can be prevented along the same lines when studied and the causes understood, and that the presence of venereal diseases today is a certain promise of their disappearance tomorrow. There is no halt in the march of science along the lines of preventive medicine, and nature's methods of elimination will be the lines along which the greatest progress will be made.

GENERAL TREATMENT.

The social evil exists from the most fallacious theories, delusions, stupid sentiments and customs, and science challenges the reasons and denies the reality of them for its presence. Most of the present means and measures to control the vice evil increase it, and in many ways promote the very conditions from which it grows, because there is no recognition of the real causes.

Exact studies show that insanity, alcoholism, idiocy, immorality and other degenerative diseases are all in the same class. The slavery and insanity of being possessed and obsessed with sexual impulses is a disease of both body and mind, demanding physical treatment.

Public sentiment formulated into laws should compel men and women to live right. Persons who become immoral and dissolute should forfeit their liberty and be regarded as incompetent to act rationally, and adjust themselves to the exact conditions of life. Such persons should be considered as dangerous and as infectious as smallpox cases and consumptives; also insane and unable to enjoy freedom of thought and conduct. They should come under legal control as violators of law and order, and be segregated in workhouse hospitals and places of refuge, where the surroundings and conditions of living can be made exact, and the morbid impulses of mind and body restricted. These hospital homes should be organized in the neighborhood of every city and town, and should constitute military schools for the confinement, medical care and training of men and women who are unable to live rationally and normally.

Such schools would receive from the courts all persons convicted of living lives of immorality and place them under exact personal care, and furnish them with occupation and duties that would make them self-supporting. These buildings should be inexpensive and situated on large tracts of land in the country, where agricultural, horticultural, mechanical and artistic industries can be carried on according to the capacity of the inmates.

The Farmfield Colony for Inebriate Women in the neighborhood of London gives an outline of what is possible to erect in every city and town in this country. This colony consists of a farm house, surrounded by small cottages and workhouses, in the center of a large tract of land. Women are housed in these cottages, according to their conditions and occupations. An attempt is made to divide them into congenial settlements. Each person is given some occupation, best suited to her taste and capacity, on the farm, in the garden, orchard, dairy, poultry yard, or looking after the cows and caring for them in a general way.

Others have duties in the laundry and kitchen, and in the workshop, where sewing and the making of garments for sale is conducted. Thus everyone has a special work to do on the farm or in the house and the workshops attached to it.

Twice a day intellectual exercises, music, lectures and every other means and measures of diversion that will build up and strengthen their higher ethical nature are furnished.

Thus homes, sanitariums and colonies of a similar character, charitable, self-supporting and under the care of the State or city, or private corporations, can be made literal hospitals for the restoration and cure of these degenerate men and women.

Such homes would enable us to ascertain causes that are not unknown and remove them. They would enable us to apply exact remedies for diseases of this kind. They would also furnish a home for the incurables and dependents, and those who are unable to care for themselves, and give to those who are not so far down an opportunity to recover.

These school hospitals in this way could concentrate and apply every possible means known to science, and, from experience, to check, restore and change these morbid impulses. They would also furnish exact fields for philanthropists, moralists and clergymen to apply and concentrate all the educative powers of sympathy and culture that will restore and give new force and power to life.

The people of the slum districts forced out of the surroundings could be placed in centers of growth and development, and made to become healthy and normal citizens. The dissolute men and women higher up will be forced to recognize the law of control, and the danger of their present condition, and use preventive measures to stop the growth of prostitution, immorality and pauperism. This is the highest teaching of modern science, and begins with coercion, positive, emphatic and literal, and segregation in the best surroundings and conditions, for self-support, change, culture, development and physical improvement.

### LEGAL TREATMENT.

Science appeals most urgently for a change of public sentiment and laws that will break up the present misconceptions and delusions of the sex evil. Scientific studies show that sexual excesses are crimes both offensive and destructive to the individual and injurious to the community; crimes against the great principles of rate continuity, law and order; crimes that mean continuous disintegration of body and mind, crimes that are suicidal and destructive, developing all sorts of degenerations, specific and general diseases, increasing mortality, precipitating old age, destitution and death.

The law is clear in its efforts to protect property and make home-life safe, and the interests of the community secure, securing liberty for the promotion of all that is good and true, but it has failed in its recognition and attempted control of the very worst form of personal criminality in prostitution, a criminality destructive to law and order and race development.

It has not yet recognized sex-obsessions and possessions as insanity. It has not recognized the irresponsibility of individuals who persist in perverting and destroying the highest purposes of life, not only for the present, but for the future. When these conditions become aggravated to an intolerable degree, a system of fines, imprisonment and perfunctory measures that are destructive and fatal to the last degree are concentrated on women, as if they were the only offenders. This is barbaric and unjust. If men received the same punishment, and were regarded as equal offenders, a change would follow.

The newer civilization calls for an equal responsibility of men and women, and the fast men of wealth, who insist on keeping mistresses, should be controlled with the same severity and exactness as the poor street-walker.

The young man who insists on a round of dissipation and who is classed among his friends as "sowing wild oats" is a criminal offender, whom the law will some day reach and control. We must recognize the teachings of science and demand that legal recognition and control of the character and conduct of men and women be equally as exact as that which concerns their property and liberty in the community.

### MEDICAL AND SURGICAL TREATMENT.

Exact scientific studies have shown the possibility of removing local palsies and irritations, and in this way sexual abnormalties may be corrected by medical and surgical treatment. Thus castration and sterilization give new direction to the morbid impulses and break up local palsies and irritations, and encourage new motives and purposes to grow and displace former impulses, and thus lift the slave of the sexual evil into the position of a freeman in the best and largest sense.

Certain surgical measures are sustained by the highest wisdom of today from the fact that they prevent the transmission of defective germs and defective race stock to the next generation. They also destroy morbid impulses and to a large degree change the direction of nerve energy and diseased obsessions. Ten States of the Union today have passed laws giving power to perform surgical operations on dements, idiots and others who are likely to transmit to the future their defects.

Notwithstanding the objections and theories which have been urged against these measures, there is a very large promise and a very large unknown field which will be occupied in the near future by surgical means and measures.

The medical care possible to be concentrated on these poor victims has scarcely been taken up for a great variety of reasons, because of the objections of public sentiment. Radium has recently been used in several experiments to diminish erotomania and sex obsessions, and the results so far are promising.

If the poor victims were housed and properly treated medically, it is impossible to say what results could be obtained. Taking women from the slum districts and confining them in jails for a few weeks or months, and sending them back to the same surroundings, is simply increasing the troubles. Thus medical treatment is practically useless, unless applied in proper surroundings with distinct control, both physical and psychical.

There is no sentiment about this. It is simply the recognition of the great fact that the social evil is a physical one that calls for physical means and measures of treatment, and the poor victim is dangerous to himself, to the community and to the State.

### SOME CONCLUSIONS.

The social evil of today is an insanity, marked by intense selfishness, dishonesty, criminality, alcoholism and retrograde standards for both the individual and the race. No plan of license or toleration in particular sections of the city is entitled to the least consideration. It is prohibition, absolute, positive and literal that furnishes the only remedy.

Immoral men and women are diseased, degenerate, sick and want help and treatment, not punishment or condemnation, but protection, and the use of practical means that will overcome this terrible obsession.

People who throw typhoid excreta on the water-shed of a city reservoir must be made to realize the terrible danger that comes from contaminated water, and be prevented from continuing this menace.

The prostitutes of today, both men and women, are practically doing the same thing. This must be recognized and positive means and measures put in force to control, segregate and prevent them from continuing and promoting this tide of degeneration.

The white slave traffic and the men and women who are promoting and encouraging this source of disease are criminals of the very worst kind, and should be arrested and housed and forced to live differently. Our modern civilization demands an elimination of this evil, and science shows the possibilities of this, through a more accurate public sentiment, materialized in law by the coercion, segregation and training of the present victims.

Beyond this, the question of breaking up the causes turns on a higher education and culture that will begin at home, extend into the school and college and dissipate the medieval theories by revealing the real purpose and object in life, and how to attain it.

We need a revolution in educational matters; we need revolution in religious conceptions. We need to realize the possibilities of human life, of culture and training and the best use we can put the resources we possess to; how we can live on the highest plane of fullest enjoyment; how we can develop the laten forces and qualities which we possess; how we can enjoy the surroundings and activities, and make life a continuous journey, free from the miseries and sufferings of the present.

There is a higher consciousness dawning in the public mind. There is a struggling of races for emancipation from the dead past. There is a foreshadowing of the coming of a new era, when men and women shall realize the real dignity and purpose of life, and there is a cosmic consciousness that disease and miseries are preventable, also that the world is the great laboratory of cause and effect, which it is our duty to study and become adapted to.

Insanity, alcoholism, criminality and sex perversions are all punishments for the violation of great laws. The great object of this congress is to make us realize what means and measures can be used for the practical solution of this great sex problem.

Science has but one voice, that that is most authortative. Science points out distinctly means and measures which, when put into actual practice, will bring about a new era for the salvation of the race.

---

Ultra-rigid asepsis and minimization of manipulation are the first great essentials in the open treatment of fractures. If these cannot be provided by the environment of the surgeon and by his experience, don't operate.—*American Journal of Surgery.*

